# Design of Bionic Cochlear Basilar Membrane Acoustic Sensor for Frequency Selectivity Based on Film Triboelectric Nanogenerator

**DOI:** 10.1186/s11671-018-2593-3

**Published:** 2018-07-03

**Authors:** Yudong Liu, Yaxing Zhu, Jingyu Liu, Yang Zhang, Juan Liu, Junyi Zhai

**Affiliations:** 1 CAS Center for Excellence in Nanoscience, Beijing Institute of Nanoenergy and Nanosystems, Chinese Academy of Sciences, Beijing, 100083 China; 2 College of Environmental Sciences and Engineering, Peking University, Beijing, China; 3 School of Nanoscience and Technology, University of Chinese Academy of Sciences, Beijing, 100049 China; 4 Center on Nanoenergy Research, School of Physical Science and Technology, Guangxi University, Nanning, 530004 China

**Keywords:** Triboelectric nanogenerator, Cochlear basilar, Acoustic sensor, Frequency selectivity

## Abstract

Sensorineural hearing loss tops the list of most suffering disease for the sake of its chronic, spirit pressing, and handicapped features, which can happen to all age groups, from newborns to old folks. Laggard technical design as well as external power dependence of conventional cochlear implant cumbers agonized patients and restrict its wider practical application, driving researchers to seek for fundamental improvement. In this paper, we successfully proposed a novel bionic cochlear basilar membrane acoustic sensor in conjugation with triboelectric nanogenerator. By trapezoidally distributing nine silver electrodes on both two polytetrafluoroethylene membranes, a highly frequency-selective function was fulfilled in this gadget, ranging from 20 to 3000 Hz. It is believed to be more discernable with the increment of electrode numbers, referring to the actual basilar membrane in the cochlear. Besides, the as-made device can be somewhat self-powered via absorption of vibration energy carried by sound, which tremendously facilitates its potential users. As a consequence, the elaborate bionic system provides an innovative perspective tackling the problem of sensorineural hearing loss.

## Background

There are many people who suffer from the hearing impediments, which caused by many reasons such as age, cancer, tuberculosis, noise, drug abuse, physical trauma worldwide [1–4]. As one of most serious and typical hearing impairment, sensorineural hearing loss is often caused by the damage or loss of hair cells of the organ of the Corti in the cochlea, which leads to the disorder of frequency discrimination of the hearing function [5–7]. The most important functions of cochlea are separating the incoming sound waves by their frequencies and convert different frequency of sound-induced vibration into electricity to stimulate auditory nerves [8, 9]. The basilar membrane which is a special film plays a significance role for the frequencies selectivity. Most of patients who suffer from the sensorineural hearing loss choose to cochlea implants, which transform the acoustic into electricity to stimulate auditory nerves through an electric array inserted in the cochlea [10, 11]. However, these cochlea implants make the patients feel very uncomfortable for they have many additional equipment located on the patients’ head, which result in many inconveniences when the patients sleep or excise. On the other hand, they also need peripheral devices to provide electric energy for all the system [12]. To overcome those disadvantages, fabricating a self-powered article and fully self-contained implantable artificial cochlea has been focus of efforts by many researchers globally.

To realize the function of frequencies selectivity like cochlea, some micro-nano structures devices have been reported. Juichi Ito and Keon Jae Lee et al. fabricated acoustic sensor which can achieve the function of frequencies selectivity based on piezoelectric materials [13–15]. However, the voltage output of these devices is relatively low which range from several microvolt to about 100 μV due to low voltage response of the piezoelectricity. On the other hand, H Shintaku et al. demonstrate an acoustic sensor fabricated of a microbeam array which only could realize the frequencies at the higher frequency when comparing with the audile one [16]. But all of these designs have some kinds of notable weaknesses, such as complex fabrication procedure of the devices, low electricity output, and frequency selection.

As a newly emerging technology field, triboelectric nanogenerator (TENG) becomes an ideal method to conquer all these problems [17–19]. Based on the coupling of electrification and electrostatic induction, marvelous electric output can be easily obtained within less expense and simple structure, avoiding sophisticated fabrication process. Such tractable mechanism/design has derived a large number of structures to facilely scavenge various kinds of mechanical energy and made self-powered device no longer a dream [20–23]. To be detailed, TENGs are essentially developed for micro- or nano-scopic mechanical-to-electrical energy conversion, which is far more compatible to the vibration of air flow and encourages a series of researches concerning it [24, 25]. For instance, by subtly absorbing acoustic energy, Yang et al. have managed to vividly record vocal print with a self-powered TENG-based microphone [26]. Note that these devices are of much sensitivity to the alternation of mechanical frequency, enlightening the advancement of next-generation frequency-selective components.

In this paper, we demonstrate a kind of acoustic device which realizes both the frequency selectivity and the transform of the acoustic energy to electrical energy. Our device is composed of two pieces of polytetrafluoroethylene (PTFE) membrane which are fixed on a trapezoidal slit located on acrylic plate, where the PTFE membrane over the slit works as sensor. The function of the polytetrafluoroethylene membrane (PM) is corresponding to the capability of nature basilar membrane, and it is successfully confirmed based on the vibration of the PM occurred at different local places according to the frequency of incoming sound waves.

## Methods/Experimental

**Fig. 1:**
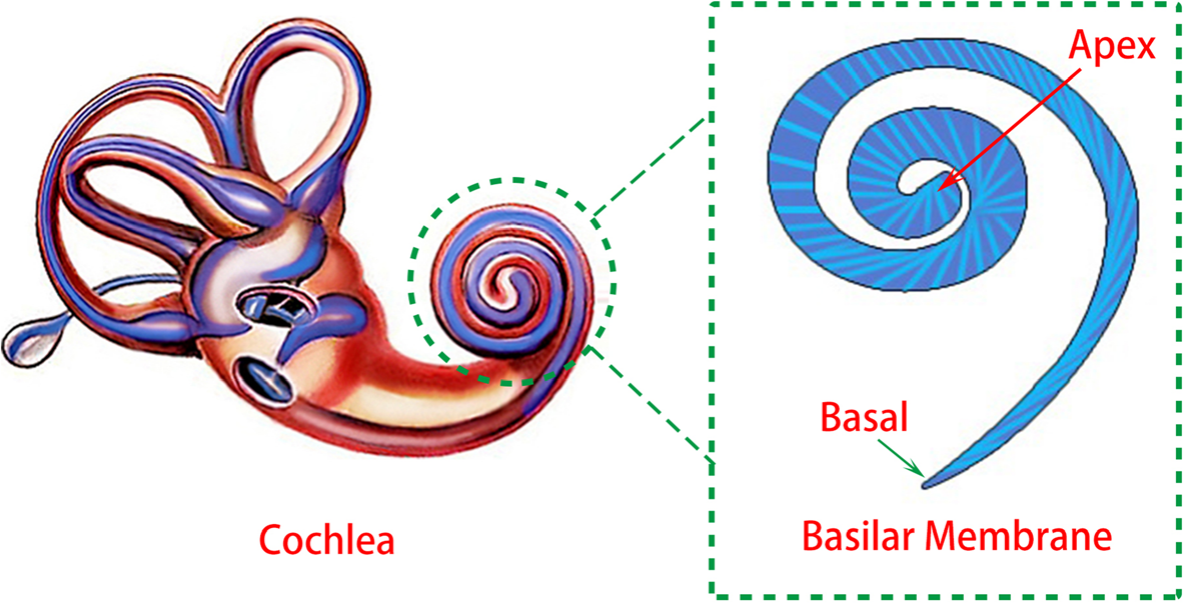
Conceptual schematics of the cochlear and the basilar membrane. The basilar membrane is a spiral thin film, the width of which was reduced gradually from the apex to the basal

Figure 1 shows the schematic drawings describing the basilar membrane of cochlear. The basilar membrane plays an important role in passive hearing [27]. Its shape is alike to a trapezoidal frame which is twisted into a spiral and covered by a thin membrane. Because of its geometric feature, the basilar membrane is capable to mechanically separate the frequency components contained in incoming acoustic waves. The apical region of basilar membrane responds to high acoustic waves, and the basal region only reacts to low-frequency sounds. When specific location of basilar membrane is vibrated by its resonance frequency acoustic wave, the hair cells lying on the membrane open or close the ion-channel to generate electrical potential [28].

**Fig. 2:**
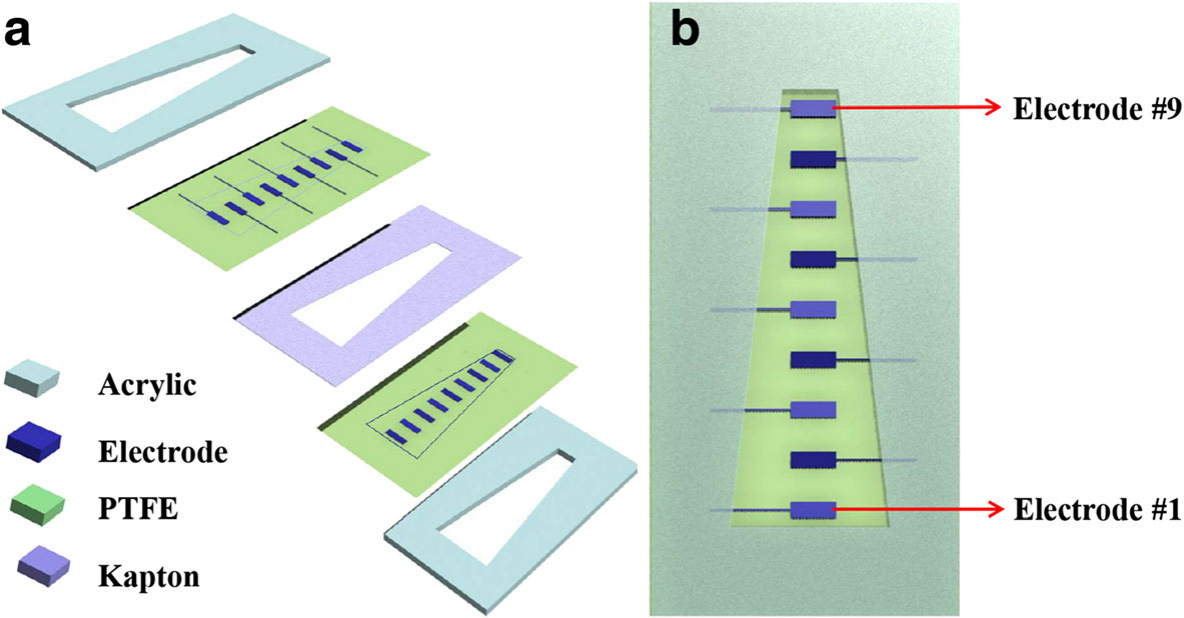
Structural design of the bionic membrane sensor. **a** The 3D view of the major components of the device for frequency selectivity. They are glued together, and only the stretchy PTFE membranes surrounded by the trapezoidal slit can vibrate freely under the sound stimulation. **b** The top view of the sensor. The electrodes, made of silver deposition, are numbered from electrode #1 to #9

The designation of membrane acoustic sensor is showed in Fig. 2. The device mainly comprises two layers of PTFE membranes, one piece of Kapton polyimide film and two pieces of acrylic plates with the trapezoidal slits. The acrylic plate is a rectangle plate, with the length of 120 mm, the width of 60 mm, and the thickness of 4 mm. The trapezoidal slit is located in the middle of the acrylic plates, and the length of baseline and topline is 30 and 10 mm, respectively, with the height of 100 mm. The PTFE membranes are similar to the acrylic plates in the length and width except that the thickness is only 20 μm. The trapezoidal shape was inspired by the cochlear basilar membrane with its local resonant frequency changes gradually from topline to baseline [29, 30]. The electrode array with nine elements made of the silver deposition is fabricated on upper side of PTFE membranes based on Magnetism Sputter System. Since the electrodes of about 200 nm thick are extremely thinner than that of PTFE (40 μm), they will not affect the vibrating characteristics of PTFE. For convenience, the electrodes are named as #1~#9 from bottom to top of the trapezoidal membrane, respectively, as shown in Fig. 2b. The size of each electrode is 4*8 mm^2^ with a rectangle shape, and the in-plane distance between two adjacent electrodes is 10 mm. The Kapton hard film, which is in the same size as the acrylic plate, is placed between the two PTFE membranes. The thickness of Kapton membrane will determine the sound pressure detection limit. The role of the Kapton film is to make a narrow gap between the two layers of the PTFE membrane. The Kapton film and PTFE membranes were strain-free covered in the middle of two acrylic plates with the trapezoidal slits with adhesive glue. The vibration of PM is measured by using a laser Doppler vibrometer measuring system (LDV) and a sound level analyzer at the various frequencies in the range from 100 to 3000 Hz. The electric signal output is measured through the electrodes using a preamplifier.

## Results and Discussion

**Fig. 3:**
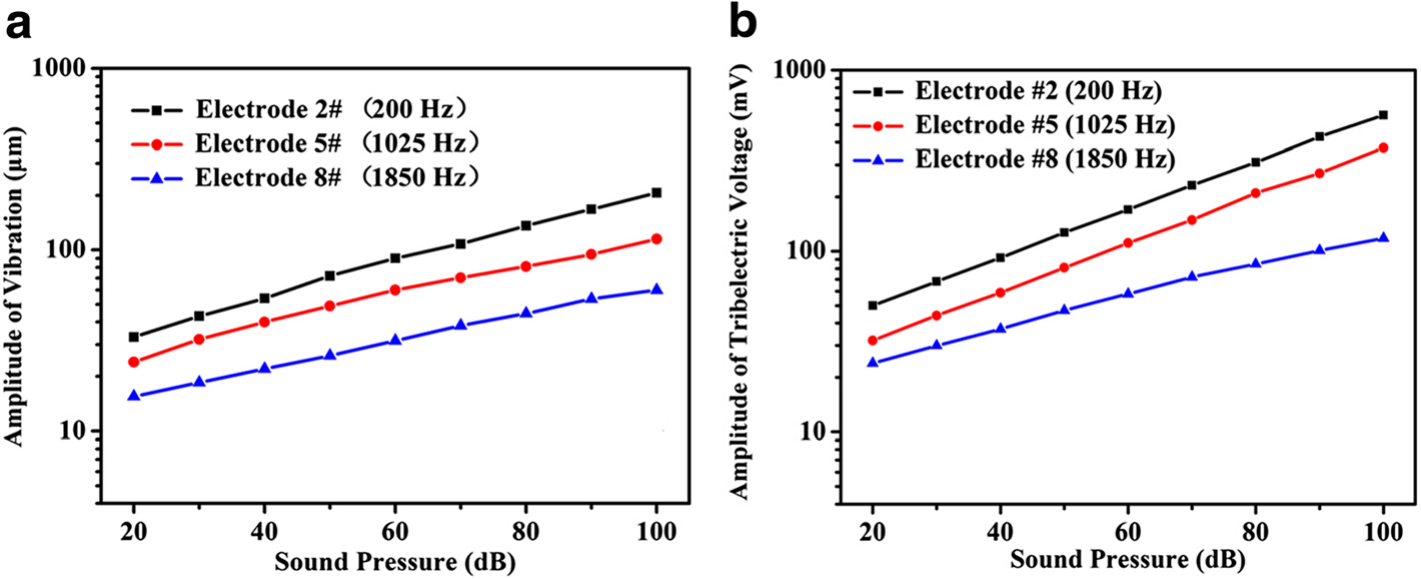
Experimental results in effect of the acoustic pressure on amplitudes **a** of the vibration and **b** of triboelectric voltage output. Apparently, it is a kind of linear relationship between amplitude and sound pressure

We first investigated the effect of sound pressure on amplitudes of vibration amplitudes of the PTFE membrane and triboelectric voltage output by LDV and oscilloscope, respectively. Figure 3 shows the relationship between the external sonic pressure and the vibration amplitude in PTFE membrane. Here, we choose the signal from the electrodes which are numbered as #2, #5, and #8. The sound pressure is provided by a speaker which could emit sinusoidal acoustic wave which was 100 mm distant from the device with a small angle at a tilt. As it can be seen in Fig. 3a, the amplitude of vibration at each electrode linearly increases with the increase of sound pressure. Also, the amplitude increase when the electrode number increased. Figure 3b shows that the relationship between the sound pressure and the amplitude of triboelectrical voltage output. The amplitude of triboelectric output also demonstrates a linear relationship with sound pressure. These results prove that the membrane acoustic sensor can detect the magnitude of acoustic wave by examining the voltage from the triboelectric nanogenerator.

**Fig. 4:**
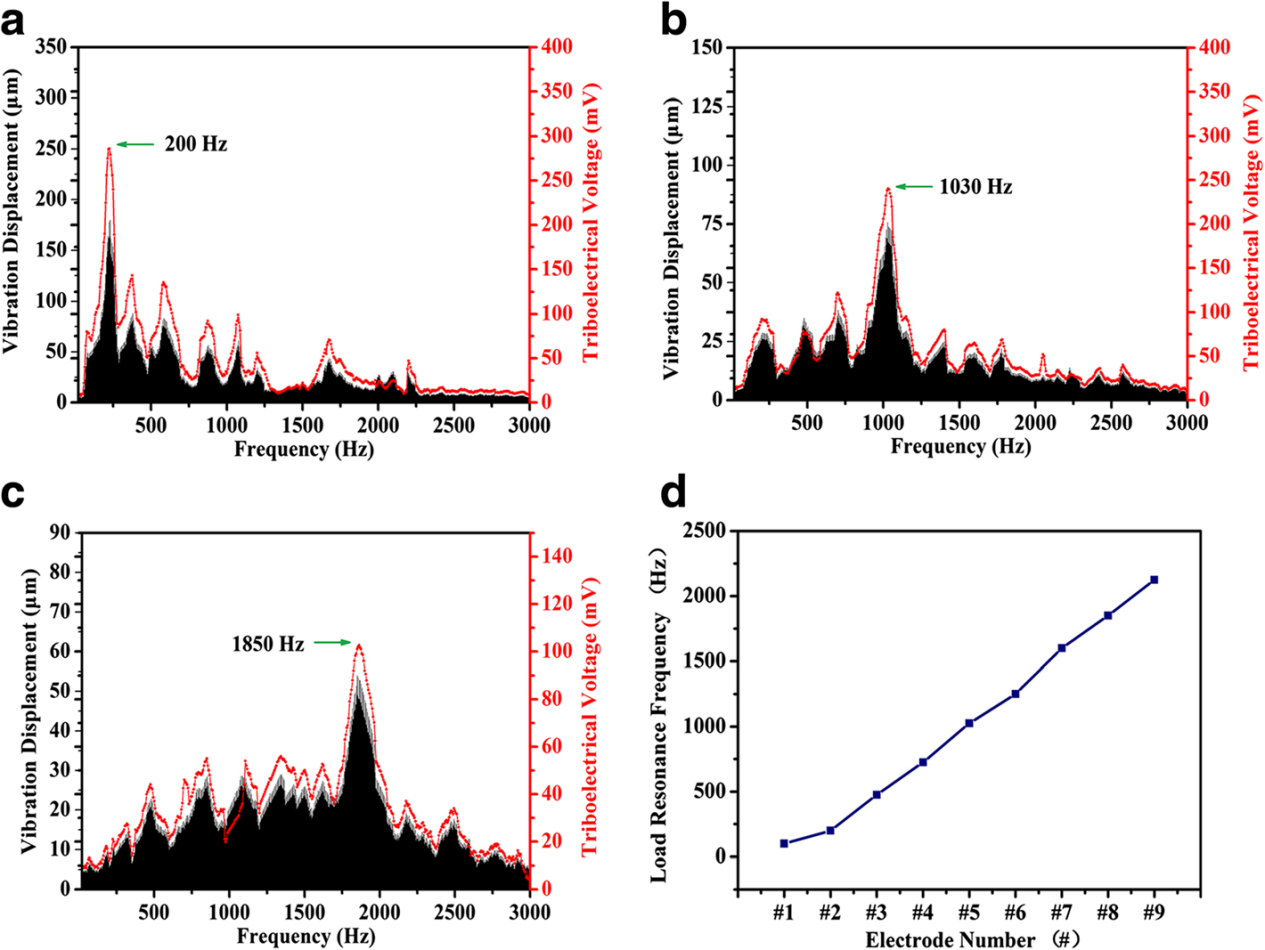
Research result of the triboelectric voltage output signal and the vibration amplitude from **a** electrode #2, **b** electrode #5, and **c** electrode #8, which was measured by LDV system and oscilloscope in the frequency ranging from 20 to 3000 Hz, and a distribution of the vibration displacement and the triboelectric voltage output signal were closely overlapped all over the frequency bandwidth. **d** Experimental results of the relationship between the electrode number and the local resonance frequency of the PTFE membrane

We next investigated the tuning capability of the membrane acoustic sensor with frequency selectivity. Figure 4a–c shows that the frequency dependence of vibration and the triboelectric voltage output at #2, #5, and #8 electrodes, respectively. The black line represents the amplitude of vibration while the output of triboelectric voltage is plotted by red line. The result shows that each electrode has a particular frequency where the electrode has relatively large outputs. The local region, where the local resonance frequency of the PTFE matches that of the incoming sound, vibrates with large amplitude result in a peak of vibration. The peak of voltage output of electrode #8 is 104 mV, which corresponds to the local region of the PTFE membrane with the peak of vibration at 1850 Hz. Analogously, the local regions with vibration amplitudes at 200 and 1030 Hz corresponded to the peak of triboelectric voltage output of electrodes #2 and #5, respectively. Besides, the frequency dependence of vibration is qualitatively similar to the triboelectric voltage output.

Figure 4d shows the relationship between the resonance frequency of local region and the electrode number. The number of electrode represents the distance from the bottom of the trapezoidal slit. Obviously, as the sound frequency increased, the peak of vibration inclined to be shift toward larger electrode number, corresponding to the base region of the actual basilar membrane in the cochlear.

**Fig. 5:**
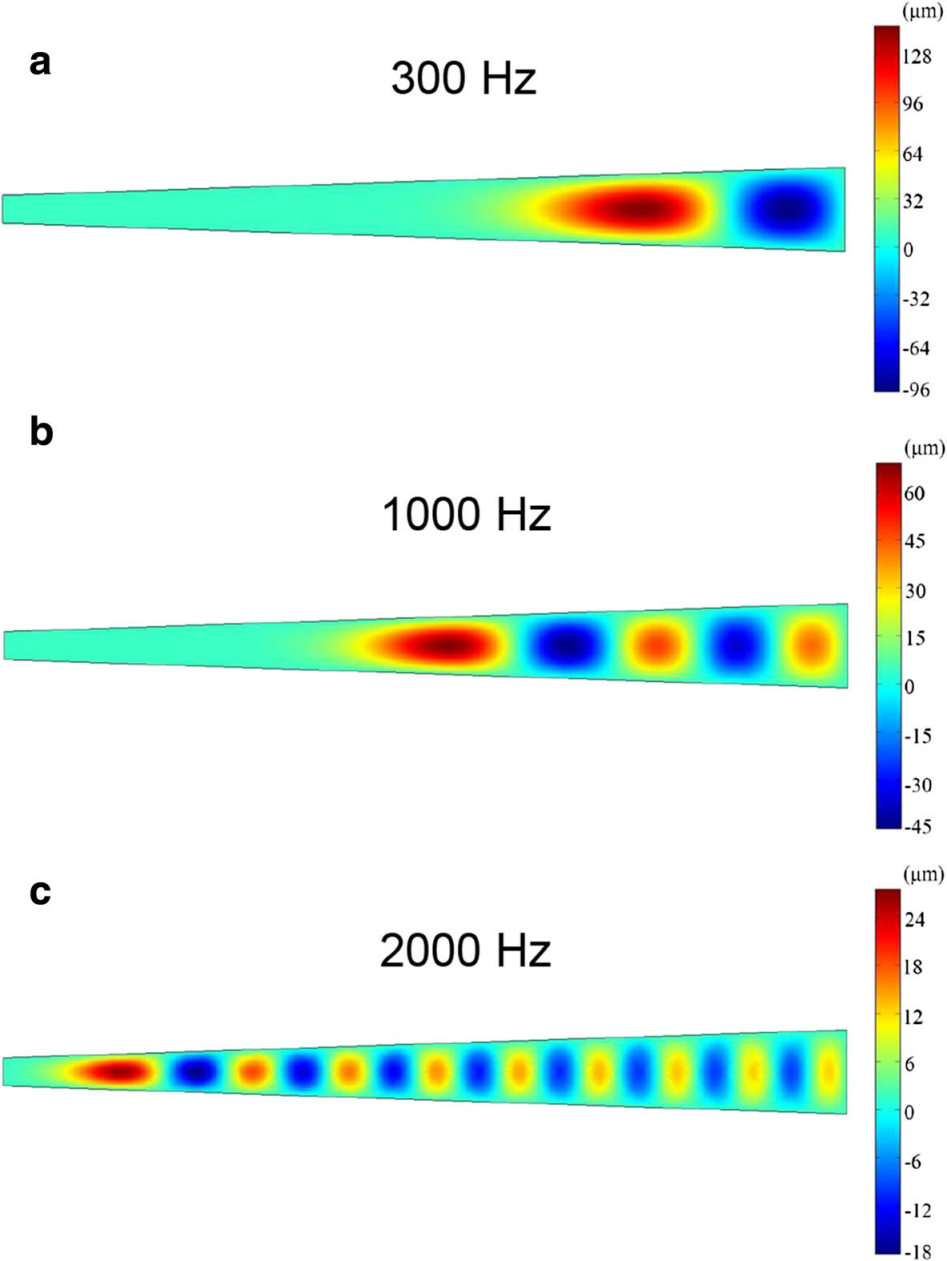
The Comsol software was employed to stimulate the vibration characteristics of single PTFE membrane at the frequency of **a** 300 Hz, **b** 1000 Hz, **c** 2000 Hz

As described earlier, the membrane acoustic sensor mimics the cochlear basilar membrane, and the operating principle can be explained by two parts, membrane acoustic vibration and vibration-induced electricity generation. On the one hand, the acoustic vibration patterns of the PTFE basilar membrane in respond to the external sound pressure at different frequencies range from 20 to 3000 Hz (the part of human’s audible frequency), were emulated by COMSOL Multiphysics, as demonstrated in Fig. 5 [31]. From the simulation result, we can find that the amplitude distribution of the PTFE membrane clearly shows dependence on the acoustic frequency. The place with the maximum amplitude, where the PTFE membrane is locally resonating, shifts from the baseline to topline of the trapezoidal area as the frequency increase, which fits the experimental results well.

**Fig. 6:**
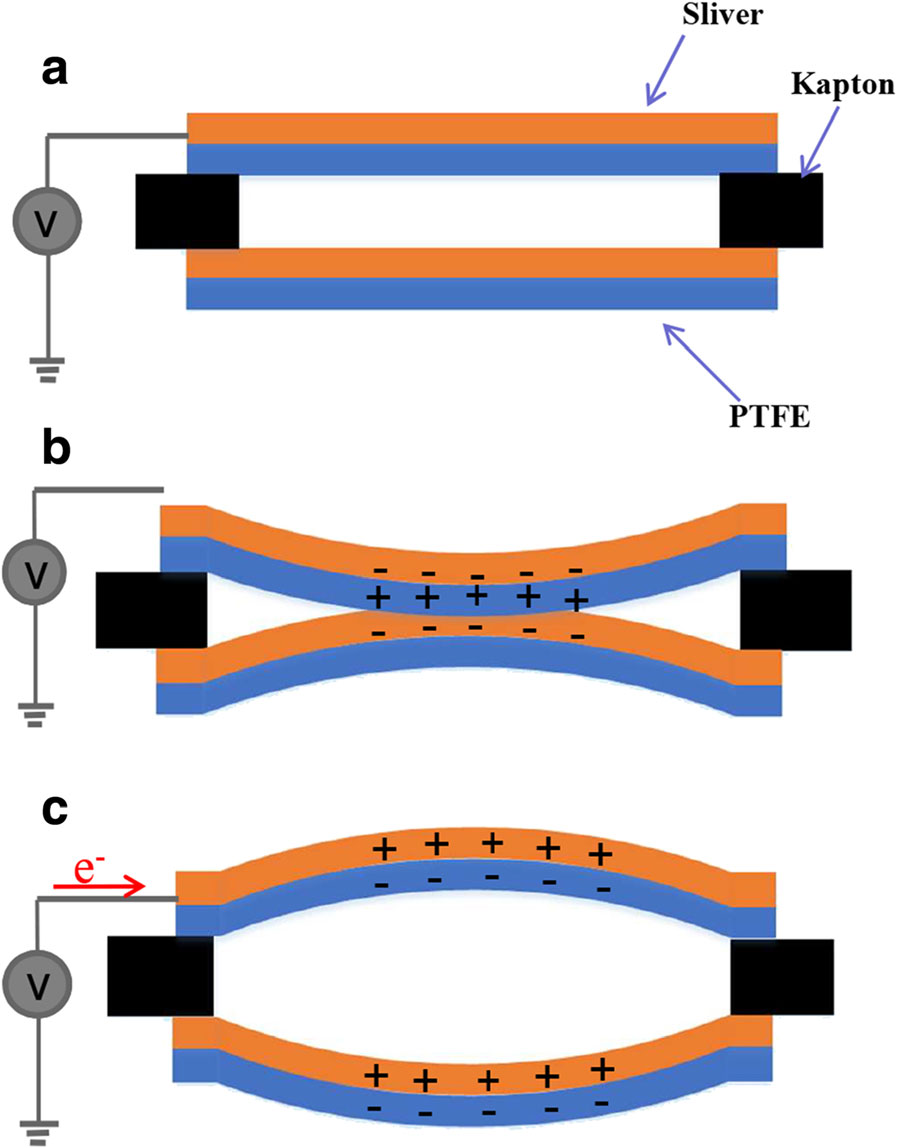
The diagram of the working principle of the sensor. **a** Rest state, in which the PTFE is not charged, without sound stimulation. **b** Contact state, in which the upper PTFE membrane is negatively charged, under sound pressure. **c** Separate state, in which the upper and lower PTFE membrane separate from each other, the potential difference drives free electrons to flow from the ground to silver electrode through the external circuit

On the other hand, the acoustic vibration of PTFE membrane-induced electricity generation is attribution to the coupling between contact electrification and electrostatic [32], as shown in Fig. 6. There is not any voltage signal when the membrane acoustic sensor is not applied by a sound (Fig. 6a). When the external sound pressure bring the upper PTFE membrane to contact with the silver deposition on the lower PTFE membrane (Fig. 6b), the PTFE grabs electrons from the silver layer, which make the negative triboelectric charges balanced by their opposite counterparts due to electrostatic induction [19]. As a result, there is neither potential difference across the two layers nor between the electronic on the upper membrane and ground. When the external sound pressure disappeared, the upper PTFE basilar membrane will bounce back from the lower PTFE membrane because of its inherent elasticity. A gap will emerge between two membrane layers (Fig. 6c), which lead the electric potential of a certain electrode drop across them owing to the triboelectric charges, the same as the relation between silver electrode and ground [33].

## Conclusions

In summary, we demonstrate a novel approach to mimic the function of basilar membrane in the cochlear which have an important effect of frequency-selectivity, by using a membrane sensor with the acoustic/electric conversion based on triboelectrific nanogenerator. The trapezoidal PTFE membrane, which was coated by several little rectangle silver electrodes, is the main component of the acoustic sensor. The vibration characteristics and the electrical signal output of trapezoidal PTFE membrane were measured by applying sound waves at a certain frequency, with the laser Doppler vibrometer and the oscilloscope. The location with the maximum amplitude was shift toward narrower area of trapezoidal PTFE membrane as the frequency increased. By this means, the sensor could realize the function of frequency selectivity. Furthermore, a finite element simulation was conducted with the COMSOL to show that the relationship between amplitude of the trapezoidal PTFE membrane and the incoming acoustic wave are fit to the experimental results. The membrane acoustic sensor demonstrates a new and effective method to solve the sensorineural hearing loss with low cost and provides an alternative to the treatment of deafness by triboelectric nanogenerator.

## Abbreviations

LDV: Laser Doppler vibrometer measuring system (LDV)
PM: Polytetrafluoroethylene membrane
PTFE: Polytetrafluoroethylene
TENG: Triboelectric nanogenerator
